# The effects of the number of consecutive night shifts on sleep duration and quality

**DOI:** 10.5271/sjweh.3885

**Published:** 2020-07-01

**Authors:** Anne Helene Garde, Kirsten Nabe-Nielsen, Marie Aarrebo Jensen, Jesper Kristiansen, Jeppe Karl Sørensen, Cand scient san publ, Åse Marie Hansen

**Affiliations:** The National Research Centre for the Working Environment, Lersø Parkallé 105, 2100 Copenhagen, Denmark; Department of Public Health, Section of Social Medicine, University of Copenhagen, Centre for Health and Society, Øster Farimagsgade 5, 1014 Copenhagen, Denmark

**Keywords:** fast rotation, night shift work, night worker, shift work, shift worker, sleep quality, slow rotation

## Abstract

**Objectives:**

The organization of night shift work affects sleep duration and quality. The aim of this study was to investigate the effects of the number of consecutive night shifts on sleep duration and quality among police officers with night shift work as part of their normal schedule.

**Methods:**

This quasi-experimental, within-subject crossover study included 73 police officers. All participants performed three work schedules: two, four and seven consecutive night shifts followed by the same number of recovery days, ie, day work or days off (2+2, 4+4, and 7+7). Sleep assessed through sleep diaries and actigraphy after all night shifts and recovery days (totaling 26 days) was compared by use of repeated measures analysis.

**Results:**

Participants experienced shorter sleep duration (with and without naps), more premature awakening, less difficulty falling asleep, and more non-refreshing sleep after night shifts compared with recovery days. Sleep duration and quality did not change with increasing number of consecutive night shifts. Sleep was shorter and of poorer quality after the last night shift in the 2+2 and 4+4 work schedule compared with the second and fourth night shift, respectively, in the 7+7 schedule.

**Conclusion:**

Sleep duration was reduced after night shift work and did not increase with more consecutive night shifts, which leads to accumulated sleep debt. Sleep duration was shortest and sleep quality was poorest after the last night shift in a series of night shifts.

It is well established that night shift work causes reduced and disturbed sleep. Night shift workers report premature awakenings and insufficient sleep, yet ~50% of individuals experience a spontaneous and effortless sleep termination (1, 2). The organization of night shifts may influence the effect of night shift work on sleep. For example, a slowly backward-rotating work schedule (eg, changing from night to evening to day shifts with seven consecutive shifts) has been found to be associated with more impairments of sleep than a fast forward-rotating work schedule (eg, changing from day to evening to night shifts with two to four consecutive shifts) (2–4). Nevertheless, only a few intervention studies have investigated the isolated effect of changing the speed of rotation (5, 6). On the one hand, these studies suggest that fast rotation, ie, few consecutive night shifts, is associated with fewer sleep disturbances and difficulties (5, 6). This would favor night shift work schedules with few consecutive night shifts. On the other hand, studies have shown that there is some adaptation of biological rhythms to sleeping during the day leading to gradual improvement of sleep with increasing consecutive night shifts (7). This would favor night shift work schedules with slow rotations, ie, more consecutive night shifts.

Short sleep duration and poor sleep quality has, in studies with longitudinal designs, been associated with higher risk of diabetes (8) and cardiovascular disease (9, 10) and may serve as a mechanism linking night shift work to increased risk of injuries and accidents (11) as well as chronic disease (12–16). When night shift work is inevitable, such as in the hospital sector and police force, it is essential to find the best way of organizing night shift work to minimize the impact of night shift work on health. To reduce unhealthy consequences of night shift work, it has been recommended to reduce the number of consecutive night shifts in order to reduce circadian disruption (17). Yet, it is, unclear what the optimal number of consecutive night shifts is when considering sleep duration and different aspects of sleep quality.

The overall aim was to contribute to the knowledge about how to optimally organize night shift work in order to reduce sleep deprivation and negative health consequences. We therefore investigated the effects of the number of consecutive night shifts on sleep duration and quality among police officers with night shift work as part of their regular schedule. During the study period, each participant was enrolled in three different work schedules: two, four and seven consecutive night shifts followed by the same number of recovery days, ie, day work or days off (hereafter: 2+2, 4+4, and 7+7). The main aim of the study was fulfilled by answering the following questions: (i) Is sleep duration shorter and sleep quality poorer after night shifts compared with recovery days (independent of work schedule)? (ii) Does sleep duration increase and sleep quality improve with an increasing number of consecutive night shifts or recovery days? (iii) Is sleep duration shorter and sleep quality poorer after the last night shift in a series of night shifts regardless of the number of previous night shifts?

## Methods

This paper presents results from the project “In the Middle of the Night”. The National Committee on Health Research Ethics in Denmark approved the study (protocol number H-4-2012-155).

### Design

Conducted with a quasi-experimental, within-subject crossover design, the study exposed the participants to three different work schedules: two night shifts followed by two recovery days (2+2), four night shifts followed by four recovery days (4+4), and seven night shifts followed by seven recovery days (7+7). The term recovery day were used for days that allowed sleep at night and included both day shifts (31%) and days off (69%). Day shifts were typically 07:00–15:00 (we allowed day shifts to end as late as 18:00) and night shifts were typically 23:00–07:00 (we allowed the start of the night shifts to vary between 22:00 and 00:00). The participants did not work any night shifts during the seven days preceding the start of the three different work schedules.

Data were collected either in the period April–June 2013 or September–November 2013. Daylight saving time began March 27 and ended October 27 in 2013. None of the participants had data collection on these two dates and only three participants collected data after the end of daylight saving time. The three different work schedules lasted 26 days in total, and they could be distributed over no longer than three months. The order of the three different work schedules occurred in the order that suited the person in charge of the personnel-on-duty planning. This person was instructed to mix the different work schedules so they occurred in different orders and to let the different work schedules in the study begin on different weekdays. In accordance with the usual way of scheduling the shifts, the starting date of the different work schedules was planned so that it also suited the individual employee. This meant that of the 64 participants who performed all three work schedules, 18 (28%) started with the 2+2, 17 (27%) with the 4+4 and 29 (45%) with the 7+7.

### Recruitment procedure and study population

Throughout all phases of the study, there was intensive collaboration and strong support from both the management in the Danish police and employee representatives from the labor union. We recruited participants from the five police districts in Zeeland, Denmark. The inclusion criteria were that the participants had to be non-smoking male police officers with night shifts as a part of their regular schedule, but not working permanent night. The officers use self-rostering to plan their normal schedules. They are therefore not regular and may vary from person to person. In most cases, they include up to four consecutive night shifts, although seven consecutive shifts were in some cases allowed if the officer requested it. Before recruitment began, information meetings were held for the leaders, the people responsible for personnel-on-duty planning, and employee representatives. All districts were also offered an initial information meeting for potential participants, which were accepted by two police districts. Thereafter, an e-mail was sent to all potential participants in all districts with an invitation to participate in the study. A total of 121 police officers showed interest in participating in the study. Of these, a total of 73 received individual, detailed information about the project either face-to-face or on the phone and completed at least one of the three work schedules, and 64 completed all three work schedules. Reasons for dropping out were holidays or other fixed duties, change to a job without night shift work or family considerations.

### Questionnaire

The participants completed a background questionnaire before starting their first work schedule in the study. From the questionnaire, we obtained information about tenure within the police force, night shift work experience, physical activity, self-rated overall health, general job satisfaction, and diurnal type.

### Sleep dairies

All participants scored their sleep quality upon awakening from their primary sleep on all 26 data collection days. Primary sleep was defined as the first sleep episode after the night shift or as sleep during the night after recovery days. Sleep was scored using a modified version of the Karolinska Sleep Diary (KSD) (18, 19). In total, seven items were used: premature awakening, difficulty falling asleep, difficulty awakening, non-refreshing sleep, disturbed sleep, number of awakenings, and overall sleep quality. Number of awakenings was given a 1–5 score (0 awakenings=1, ≥4 awakenings=5). All other items were scored on a 5-point scale, with higher scores representing poorer sleep. In their sleep dairies, participants also noted the number, timing, and duration of naps.

### Actigraphy

Actiwatches (ActiGraph wGT3X-BT from ActiGraph FL, USA) were worn on the non-dominant wrist during all 26 data collection days. Data were collected with a sampling rate of 30 Hz and 1-minute epochs were used to score sleep. Data were analyzed with ActiGraph Sleep Analysis (ActiGraph, FL, USA). In and out of bed times were taken from the sleep dairies. The following variables were extracted: primary sleep duration (PSD), total sleep time per day including naps (TST), and sleep efficiency.

### Statistical analysis

Unless otherwise stated, we performed repeated measures ANOVA using the PROC MIXED procedure with a random intercept for each individual to account for within subject variation (20). Separate analyses were made with ten parameters of sleep duration and quality as continuous outcomes: premature awakening, difficulty falling asleep, difficulty awakening, non-refreshing sleep, disturbed sleep, number of awakenings and sleep quality from sleep diaries as well as PSD, TST and sleep efficiency from actigraphy. The MIXED procedure has the advantage that it can accommodate data that are missing at random. Due to multiple statistical testing, results were considered to be statistically significant at P<0.001.

In the first set of analyses, sleep duration and quality after night shifts were compared with sleep duration and quality on recovery days independent of work schedule using the full dataset (all night shifts and recovery days, totaling 26 days) (research question 1). In the second set of analyses, repeated measures linear regression analysis was used to evaluate if there was a change in sleep duration and quality with increasing number of consecutive night shifts or recovery days (research question 2). These analyses were performed separately for night shifts and recovery days. In this set of analyses, the last night shift and the last recovery day of each work schedule were excluded, ie, the second night shift and the second recovery day in 2+2 and the fourth night shift and the fourth recovery day in 4+4 etc. This was done because (later) analyses showed that sleeping behavior was different after the last night shift, and including these data in the analysis could mislead the interpretation of the effect of number of consecutive of night shifts on sleep. In the third set of analyses, we investigated if sleep duration and quality after the last night shift in a series of night shifts differed from other days (research question 3). We compared sleep duration and quality after the last night shift in the 2+2 and 4+4 work schedule with the second and fourth night shift, respectively, in the 7+7 work schedule (reference). All statistical analyses were done in the statistical software SAS for Windows 9.4 (TS level 1M3, SAS Institute, Cary, NC, USA).

## Results

The participants were aged 25–62 years with a mean age of 38 years; 22% had <3 years of night shift work experience, 38% had 3–10 years of night shift work experience, and 40% had >10 years of night shift work experience. Most participants rated their health excellent (31%) or very good (47%) and 78% were moderately or highly physically active in their leisure time (table 1). Of the participants, <9% worked during the day on the first recovery day after a series of night shifts. On average, 38% of the recovery days (excluding the first recovery day) were (daytime) work days and 62% were days off. Of the 2+2 work schedule recovery days, 13% were (daytime) work days, whereas 35% and 34% of recovery days in the 4+4 and 7+7 schedules, respectively, were (daytime) work days. The average awakening time was 06:48 hours on recovery days with work and 07:25 hours on recovery days without work.

**Table 1:** Description of 73 participating police officers. [SD=standard deviation.]

	N	%	Mean (SD)	Range
Age (years)	73		38 (10)	25–62
Tenure within the police force (years)	73		11 (10)	1–32
Night shift work experience (years)			11 (8)	1–30
<3	16	22		
3–10	27	38		
>10	29	40		
Physical activity				
Physically inactive	3	4		
Light	13	18		
Moderate	34	47		
High	22	31		
Self-rated overall health				
Excellent	22	31		
Quite good	34	47		
Good	14	19		
Less good	2	3		
Poor	0	0		
General job satisfaction				
Very dissatisfied	3	4		
Dissatisfied	0	0		
Satisfied	29	40		
Very satisfied	41	56		
Diurnal type				
Morning	10	14		
More morning than evening	10	14		
More evening than morning	38	52		
Evening	15	21		

**Figure 1:**
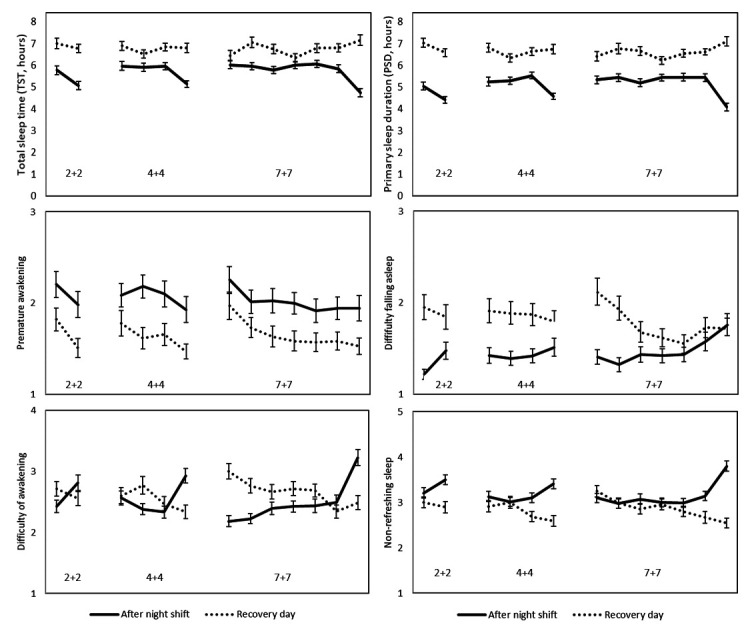
Average (standard error of the mean) sleep duration and quality on three different work schedules (2+2, 4+4, and 7+7).

Figure 1 illustrates PSD, TST, premature awakening, difficulty falling asleep, difficulty awakening, and non-refreshing sleep for all 26 days in the 2+2, 4+4, and 7+7 schedules. Data for all variables of sleep duration and sleep quality for all 26 days in the three different work schedules are shown in the supplementary material (www.sjweh.fi/show_abstract.php?abstract_id=3885 table S1). PSD was 01:32 [standard deviation (SD) 00:04] hours and TST was 01:04 (SD 00:04) hours shorter after night shifts compared with recovery days (table 2) (research question 1). The officers also reported more premature awakening, less difficulty falling asleep and more non-refreshing sleep after night shifts compared with recovery days (table 2). Table 2 also shows the estimated slope in sleep duration and quality with an increasing number of consecutive night shifts or recovery days, respectively (research question 2). Sleep duration and quality did not change with increasing number of consecutive night shifts. In contrast, difficulty falling asleep, difficulty awakening, non-refreshing sleep, disturbed sleep, and number of awakenings decreased with more consecutive recovery days (P≤0.001).

**Table 2:** Sleep duration and quality after night shifts and recovery days. Statistically significant results are shown in bold. [SEM= standard error of the mean; SD=standard deviation; PSD=primary sleep duration; TST=total sleep time (including naps)].

	After night shifts				Recovery days				Difference (diff) between night and recovery day (ref)			P-value	
			
	Average	SEM	Slope ^a^	SD	Average	SEM	Slope ^a^	SD	Diff. ^b^	SD	P-value	Slope – night ^a^	Slope – rec day ^a^
PSD ^c^ (h:mm)	**5:07**	**0:03**	0:03	00:01	**6:39**	**0:03**	0:02	0:02	**-1:32**	**0:04**	**<0.001**	0.038	0.232
TST ^c^ (h:mm)	**5:43**	**0:03**	0:00	00:01	**6:47**	**0:04**	0:00	0:02	**-1:04**	**0:04**	**<0.001**	0.760	0.712
Premature awakening ^d^	**2.0**	**0.04**	-0.054	0.02	**1.7**	**0.03**	-0.060	0.02	**0.39**	**0.04**	**<0.001**	0.011	0.003
Difficulty falling asleep ^d^	**1.4**	**0.02**	0.036	0.01	**1.8**	**0.03**	**-0.083**	**0.02**	**-0.37**	**0.04**	**<0.001**	0.007	**<0.001**
Difficulty of awakening ^d^	2.5	0.03	0.021	0.02	2.6	0.03	**-0.063**	**0.02**	-0.10	0.04	0.008	0.189	**0.001**
Non-refreshing sleep ^d^	**3.2**	**0.03**	-0.013	0.02	**2.9**	**0.03**	**-0.069**	**0.02**	**0.32**	**0.04**	**<0.001**	0.413	**<0.001**
Disturbed sleep ^d^	1.9	0.03	-0.046	0.02	1.9	0.03	**-0.086**	**0.02**	-0.03	0.04	0.465	0.006	**<0.001**
Awakenings (number) ^d^	2.5	0.04	-0.041	0.02	2.5	0.04	**-0.077**	**0.02**	0.03	0.05	0.504	0.054	0.001
Sleep quality ^d^	2.2	0.03	-0.033	0.02	2.1	0.03	-0.057	0.02	0.08	0.04	0.026	0.031	0.003
Efficiency ^c^ (%)	88.1	0.28	-0.19	0.13	87.4	0.27	0.01	0.14	0.66	0.32	0.040	0.142	0.950

a Analyses are performed without the last day.

b Estimated difference between night shift and recovery days (reference).

c Assessed from actigraphy.

d Assessed from sleep dairies (response categories: 1-5 with higher number representing poorer sleep).

Participants had shorter PSD (B= -01:01, SD 0:13 hours) and TST (B= -00:53, SD 0:14 hours) after the last night shift in the 2+2 work schedule compared with the second night shift in the 7+7 work schedule (research question 3). Participants also experienced more difficulty awakening (B=00.58, SD 00.13 hours) and more non-refreshing sleep (B=00.51, SD 00.11 hours) after the last night shift in the 2+2 work schedule compared with the second night shift in the 7+7 work schedule. Similar differences were observed after the last night shift in the 4+4 schedule compared with the fourth night shift in the 7+7 work schedule (table 3). There were no other statistically significant differences in this set of analyses. The cumulative sleep debt can be estimated as: (number of consecutive nights) × (difference between TST on night shift and recovery days) + (the difference between the last night shift in a series and other night shifts). As an example, the estimate for two consecutive night shifts is: two consecutive night shifts × 01:04 hours per night shift + 00:53 hours equaling 03:01 hours. Thus, the estimated cumulative sleep loss is 03:01, 05:09, and 08:21 hours after two, four and seven consecutive night shifts, respectively.

**Table 3:** The estimated difference of sleep duration and quality after the last night shift in the 2+2 and 4+4 schedules compared to the second and fourth night shift in the 7+7 schedule. Statistically significant results are shown in bold. N=73 police officers, who performed all three different work schedules in the study. [Diff=difference; SD=standard deviation; PSD=primary sleep duration; TST=total sleep time (including naps)]

	2+2 ^a^			4+4 ^a^		
	
	Diff.	SD	P-value	Diff.	SD	P-value
PSD (h:mm) ^b^	**1:01**	**0:13**	**<0.001**	**0:51**	**0:10**	**<0.001**
TST (h:mm) ^b^	**0:53**	**0:14**	**<0.001**	**0:53**	**0:11**	**<0.001**
Premature awakening ^c^	-0.03	0.16	0.831	-0.08	0.17	0.650
Difficulty falling asleep ^c^	0.15	0.11	0.159	0.09	0.12	0.447
Difficulty of awakening ^c^	**0.58**	**0.13**	**<0.001**	**0.51**	**0.12**	**<0.001**
Non-refreshing sleep ^c^	**0.51**	**0.11**	**<0.001**	**0.42**	**0.11**	**<0.001**
Disturbed sleep ^c^	-0.06	0.12	0.643	-0.09	0.14	0.530
No. of awakenings ^c^	-0.22	0.17	0.197	-0.36	0.19	0.057
Sleep quality ^c^	-0.12	0.12	0.323	-0.04	0.13	0.779
Sleep efficiency ^b^ (%)	-0.56	0.92	0.545	-0.68	0.99	0.494

a Data from ‘last night shifts’ are presented for 2+2 and 4+4 schedule. The second night shift in 2+2 is compared to the second night shift in the 7+7 schedule (reference). The fourth night shift in 4+4 is compared to the fourth night shift in 7+7 (reference).

b Assessed from actigraphy.

c Assessed from sleep dairies (response categories: 1-5 with higher number representing poorer sleep).

## Discussion

In this study of 73 police officers working 2+2, 4+4 and 7+7 work schedules, we found that participants experienced shorter sleep duration (with and without naps), more premature awakening, less difficulty falling asleep, and more non-refreshing sleep after night shifts compared with recovery days. Sleep duration and quality did not change with increasing number of consecutive night shifts, but reports of difficulty falling asleep, difficulty awakening, non-refreshing sleep, and disturbed sleep decreased with increasing number of consecutive recovery days. Sleep was shorter and of poorer quality after the last night shift in a series of night shifts.

It has been argued that more consecutive night shifts cause adaptation of circadian rhythms leading to better and longer sleep during the day eg, among employees on oil rigs in the North Sea (7). In contrast, the results of the present study clearly showed that sleep duration and quality did not change with number of consecutive night shifts among night shift workers. Therefore, adaptation of sleep duration did not occur, and the participants slept at least one hour less after a night shift than on recovery days even after six consecutive night shifts.

We have previously shown results regarding diurnal rhythms of hormones from the same study (21). The rhythm of melatonin was suppressed and cortisol was phase delayed with increasing number of consecutive night shifts, and after six consecutive night shifts there was not a full adaptation of the diurnal rhythms to day time sleep. In total, neither the diurnal rhythms of hormones nor the sleep duration fully adapted to night shifts. Accordingly, the police officers build up sleep debt with more consecutive night shifts. While alertness appears rather robust against small curtailments of sleep (1), experimental laboratory studies have shown that as little as two hours of daily sleep restriction over two weeks leads to accumulative decline in cognitive performance (22).

Previous studies have found that few consecutive night shifts (as part of a fast forward-rotating schedules) were associated with fewer sleep disturbances and difficulties than more consecutive night shifts (as part of a slowly backwards-rotating schedules) (2–6). In the present study, we specifically studied the effects of the number of consecutive night shifts without changing the direction of rotation, and found that participants had particularly short and poor sleep after the last night shift after both two and four consecutive shifts. Particularly, the participants reported more difficulty awakening and more non-refreshing sleep after the last night shift in a series of night shifts. This finding is in accordance with the notion that shift workers shorten their sleep after the last night shift in order to change back to night-time sleep. Thus, if an employee has to cover a fixed number of night shifts, the employee will have more “last night shifts” in a schedule with few consecutive night shifts compared with a schedule with more consecutive night shifts. Therefore, a schedule with few consecutive night shifts (fast rotation) implies more days with poor sleep compared with a schedule with more consecutive night shifts.

Our findings – that night shift workers experience shorter sleep and find it easier to fall asleep and wake up although not feeling refreshed after night shift work – are congruent with previous studies (1, 2). The total time awake when working the first night shift is often 20–22 hours or more, whereas it is 16–18 hours for a worker with permanent day work. Thus, the sleep pressure is higher after the first night shift, which may explain why participants experience less difficulty falling asleep after a night shift compared with a recovery day. Furthermore, night workers sleep during the day and typically wake up around midday, where experienced sleepiness is lower than in the morning due to circadian rhythms (23). This may explain why the participants experience less difficulty awakening after night shifts compared with day work or days off despite having shorter sleep duration and not feeling refreshed.

### Strengths and limitations

The study comprised a relatively large number of participants compared with previous field studies. In combination with the quasi-experimental (rather than observational) cross-over design and taking within subject variation into account in the statistical analyses, this is a strength of the study. We thereby enhanced internal validity by circumventing potential confounding from, eg, age and lifestyle factors, and accordingly we did not include these variables in the statistical analyses. The study was performed among experienced shift workers in a real-life setting, which enhances the external validity of the results. To avoid the risk of false positive findings due to multiple statistical testing, we considered P<0.001 to indicate statistical significance.

Another strength was the handling of the general limitations with cross-over designs, ie, possible order effects and carry-over between the different work schedules in the study. The order effect was limited by instructing the schedule planner to mix the different work schedules so they occurred in different orders, which resulted in 28%, 27%, and 45% of participants starting with 2+2, 4+4, or 7+7 work schedules, respectively. Carry-over effects were minimized by having a seven-day wash-out period without night shift work before the first night shift in a series. For practical reasons and in order to resemble real-life scheduling, recovery days were allowed to include both days off and day shifts. The rationale was that both are day-time oriented as opposed to days with night shifts and because this is the standard way to schedule night shift work among the police in Denmark. Since the normal day shift for police officers begin at 07:00 hours, it may be argued that police officers need to get up before their natural wake up time of days with day shift and that differences in the proportion of day shifts and days off would therefore bias the results. However, the bias is expected to be limited as there was a fair distribution of day shifts across the different work schedules (particularly 4+4 and 7+7) and awakening time on day shifts and days off differed by only 37 minutes.

Each work schedule was only performed once. As a consequence, it was only possible to study acute effects and not effects of long-term scheduling. However, effects of night shift work on sleep are generally acute and reversible, and we expect that the main potential differences between the three different work schedules in relation to sleep are covered with the present design. The study population consisted of male shift workers. Accordingly, we cannot be sure if the results extend to women, although similar biological mechanisms are likely. It may be speculated that permanent night work allows for better adaptation to daytime sleep because permanent night workers do not have to change their diurnal rhythms between night shifts (ie, on their days off). However, even permanent night workers are likely to being awake during the days on days off, eg, to be with family and for social reasons. Indeed, it has been shown that only a very small minority (0.3%) of permanent night workers show “complete” adjustment of their endogenous melatonin rhythm to night work (24). Yet, future research should address sleep and diurnal rhythms among workers with permanent night work.

In conclusion, we found that sleep duration was reduced after night shift work and did not increase with more consecutive night shifts. This leads to accumulated sleep debt with more consecutive night shifts. Furthermore, sleep duration was shortest and sleep quality particularly poor after the last night shift in a series of night shifts.

## Supplementary material

Supplementary material

## References

[1] Akerstedt T (2003). Shift work and disturbed sleep/wakefulness. Occup Med (Lond).

[2] Sallinen M, Kecklund G (2010). Shift work, sleep, and sleepiness - differences between shift schedules and systems. Scand J Work Environ Health.

[3] Bambra CL, Whitehead MM, Sowden AJ, Akers J, Petticrew MP (2008). Shifting schedules:the health effects of reorganizing shift work. Am J Prev Med.

[4] Driscoll TR, Grunstein RR, Rogers NL (2007). A systematic review of the neurobehavioural and physiological effects of shiftwork systems. Sleep Med Rev.

[5] Williamson AM, Sanderson JW (1986). Changing the speed of shift rotation:a field study. Ergonomics.

[6] Hornberger S, Knauth P (1998). Follow-up intervention study on effects of a change in shift schedule on shiftworkers in the chemical industry. Int J Ind Ergon.

[7] Bjorvatn B, Stangenes K, Oyane N, Forberg K, Lowden A, Holsten F (2006). Subjective and objective measures of adaptation and readaptation to night work on an oil rig in the North Sea. Sleep.

[8] Cappuccio FP, D'Elia L, Strazzullo P, Miller MA (2010). Quantity and quality of sleep and incidence of type 2 diabetes:a systematic review and meta-analysis. Diabetes Care.

[9] Cappuccio FP, Cooper D, D'Elia L, Strazzullo P, Miller MA (2011). Sleep duration predicts cardiovascular outcomes:a systematic review and meta-analysis of prospective studies. Eur Heart J.

[10] Aziz M, Ali SS, Das S, Younus A, Malik R, Latif MA (2017). Association of Subjective and Objective Sleep Duration as well as Sleep Quality with Non-Invasive Markers of Sub-Clinical Cardiovascular Disease (CVD):A Systematic Review. J Atheroscler Thromb.

[11] Wagstaff AS, Sigstad Lie JA (2011). Shift and night work and long working hours--a systematic review of safety implications. Scand J Work Environ Health.

[12] Hansen AB, Stayner L, Hansen J, Andersen ZJ (2016). Night shift work and incidence of diabetes in the Danish Nurse Cohort. Occup Environ Med.

[13] Kivimäki M, Batty GD, Hublin C (2011). Shift work as a risk factor for future type 2 diabetes:evidence, mechanisms, implications, and future research directions. PLoS Med.

[14] Stevens RG, Brainard GC, Blask DE, Lockley SW, Motta ME (2014). Breast cancer and circadian disruption from electric lighting in the modern world. CA Cancer J Clin.

[15] Torquati L, Mielke GI, Brown WJ, Kolbe-Alexander T (2018). Shift work and the risk of cardiovascular disease. A systematic review and meta-analysis including dose-response relationship. Scand J Work Environ Health.

[16] Vyas MV, Garg AX, Iansavichus AV, Costella J, Donner A, Laugsand LE (2012). Shift work and vascular events:systematic review and meta-analysis. BMJ.

[17] Bonde JP, Hansen J, Kolstad HA, Mikkelsen S, Olsen JH, Blask DE (2012). Work at night and breast cancer--report on evidence-based options for preventive actions. Scand J Work Environ Health.

[18] Akerstedt T, Knutsson A, Westerholm P, Theorell T, Alfredsson L, Kecklund G (2002). Sleep disturbances, work stress and work hours:a cross-sectional study. J Psychosom Res.

[19] Keklund G, Åkerstedt T (1997). Objective components of individual differences in subjective sleep quality. J Sleep Res.

[20] Moser EB Repeated Measures Modeling With PROC MIXED SAS proceedings, paper 188-29.

[21] Jensen MA, Hansen AM, Kristiansen J, Nabe-Nielsen K, Garde AH (2016). Changes in the diurnal rhythms of cortisol, melatonin, and testosterone after 2, 4, and 7 consecutive night shifts in male police officers. Chronobiol Int.

[22] Van Dongen HP, Maislin G, Mullington JM, Dinges DF (2003). The cumulative cost of additional wakefulness:dose-response effects on neurobehavioral functions and sleep physiology from chronic sleep restriction and total sleep deprivation. Sleep.

[23] Gradisar M, Lack L (2004). Relationships between the circadian rhythms of finger temperature, core temperature, sleep latency, and subjective sleepiness. J Biol Rhythms.

[24] Folkard S (2008). Do permanent night workers show circadian adjustment?A review based on the endogenous melatonin rhythm. Chronobiol Int.

[25] Nabe-Nielsen K, Jensen MA, Hansen AM, Kristiansen J, Garde AH (2016). What is the preferred number of consecutive night shifts?results from a crossover intervention study among police officers in Denmark. Ergonomics.

