# Supplementary material for: The effects of the number of consecutive night shifts on sleep duration and quality
Source: Scand J Work Environ Health. 2020 Jul 1;46(4):446–53. doi: 10.5271/sjweh.3885 (PMC8527589; doi:10.5271/sjweh.3885)
Supplement: Supplementary material [file SJWEH-46-446-S001.pdf]

The effects of the number of consecutive night shifts on sleep duration and quality[1]

by Anne Helene Garde, PhD,[2] Kirsten Nabe-Nielsen, PhD, Marie Aarrebo Jensen, PhD, Jesper Kristiansen, PhD, Jeppe Karl Sørensen, Cand scient san publ, Åse Marie Hansen, PhD, MD

1. *Supplementary table*
2. *Correspondence to: Anne Helene Garde, The National Research Centre for the Working Environment, Lersø Parkallé 105, 2100 Copenhagen, Denmark. [E-mail: ahg@nfa.dk]*

Supplementary table 1. Sleep duration and quality.

Inter-vention	Night/ Rec.	Day	PSD (h:mm)	TST (h:mm)	Premature awakening	Difficulty falling asleep	Difficulty of awakening	Non-refreshing sleep	Disturbed sleep	No. of awakenings	Sleep quality	Sleep onset (hh:mm)
			Avg (SD)	Avg (SD)	Avg (SD)	Avg (SD)	Avg (SD)	Avg (SD)	Avg (SD)	Avg (SD)	Avg (SD)	Avg (SD)
2+2	Night	1	05:03 (1:25)	05:46 (1:37)	2.2 (1.2)	1.2 (0.5)	2.4 (0.8)	3.2 (0.9)	1.9 (0.9)	2.5 (1.1)	2.2 (0.8)	89 6,3
2+2	Night	2	04:25 (1:16)	05:04 (1:30)	2.0 (1.2)	1.5 (0.7)	2.8 (1.0)	3.5 (0.9)	1.8 (0.9)	2.2 (1.1)	2.2 (0.9)	89 6,6
2+2	Rec.	1	07:02 (1:36)	07:00 (1:50)	1.8 (1.0)	2.0 (1.1)	2.7 (1.0)	3.0 (0.9)	2.0 (1.0)	2.7 (1.3)	2.2 (1.0)	88 7,5
2+2	Rec.	2	06:35 (1:20)	06:46 (1:28)	1.5 (0.8)	1.8 (1.1)	2.6 (0.9)	2.9 (1.0)	1.8 (0.7)	2.2 (1.1)	2.1 (0.9)	87 8,0
4+4	Night	1	05:15 (1:37)	05:58 (1:44)	2.1 (1.1)	1.4 (0.7)	2.6 (1.0)	3.1 (0.9)	1.9 (0.9)	2.8 (1.0)	2.3 (0.8)	88 9,5
4+4	Night	2	05:18 (1:21)	05:54 (1:32)	2.2 (1.0)	1.4 (0.7)	2.4 (0.8)	3.0 (0.8)	1.8 (0.8)	2.6 (1.2)	2.1 (0.7)	88 7,8
4+4	Night	3	05:32 (1:16)	05:57 (1:21)	2.1 (1.2)	1.4 (0.6)	2.3 (0.8)	3.1 (0.9)	2.0 (1.0)	2.8 (1.2)	2.2 (0.8)	88 7,1
4+4	Night	4	04:35 (1:09)	05:08 (1:18)	1.9 (1.2)	1.5 (0.8)	2.9 (1.0)	3.4 (0.9)	1.7 (0.9)	2.2 (1.2)	2.1 (0.9)	87 7,4
4+4	Rec.	1	06:49 (1:39)	06:53 (1:37)	1.8 (1.1)	1.9 (1.1)	2.6 (1.1)	2.9 (1.0)	2.0 (1.1)	2.7 (1.4)	2.3 (1.1)	88 8,3
4+4	Rec.	2	06:20 (1:33)	06:31 (1:33)	1.6 (1.0)	1.9 (1.1)	2.8 (1.2)	3.0 (1.1)	2.1 (1.0)	2.5 (1.3)	2.2 (1.0)	85 9,1
4+4	Rec.	3	06:38 (1:25)	06:50 (1:29)	1.7 (1.0)	1.9 (1.0)	2.5 (1.0)	2.7 (0.9)	1.7 (0.9)	2.4 (1.3)	2.1 (0.9)	87 6,8
4+4	Rec.	4	06:45 (1:44)	06:48 (1:43)	1.5 (0.7)	1.8 (1.0)	2.3 (0.9)	2.6 (1.0)	1.7 (0.8)	2.3 (1.1)	1.9 (0.7)	87 11,5
7+7	Night	1	05:20 (1:29)	06:01 (1:27)	2.3 (1.2)	1.4 (0.7)	2.2 (0.8)	3.1 (0.9)	2.1 (0.9)	2.7 (1.2)	2.3 (0.9)	88 7,1
7+7	Night	2	05:26 (1:25)	05:57 (1:22)	2.0 (1.1)	1.3 (0.6)	2.2 (0.7)	3.0 (0.8)	1.8 (0.8)	2.4 (1.2)	2.3 (0.8)	90 6,2
7+7	Night	3	05:12 (1:28)	05:47 (1:19)	2.0 (1.1)	1.4 (0.7)	2.4 (0.9)	3.1 (1.0)	2.0 (0.9)	2.5 (1.2)	2.4 (0.8)	89 7,2
7+7	Night	4	05:26 (1:19)	06:00 (1:24)	2.0 (1.0)	1.4 (0.6)	2.4 (0.7)	3.0 (0.8)	1.8 (0.8)	2.6 (1.2)	2.2 (0.8)	88 8,5

7+7	Night	5	05:27	(1:29)	06:04	(1:21)	1.9	(1.1)	1.4	(0.7)	2.4	(0.9)	3.0	(0.8)	1.7	(0.9)	2.4	(1.1)	2.1	(0.7)	89	6,9
7+7	Night	6	05:26	(1:26)	05:50	(1:28)	1.9	(1.0)	1.6	(0.9)	2.5	(1.0)	3.1	(0.9)	1.7	(0.7)	2.5	(1.2)	2.1	(0.8)	87	8,7
7+7	Night	7	04:05	(1:25)	04:44	(1:39)	1.9	(1.2)	1.8	(1.0)	3.2	(1.1)	3.8	(1.0)	1.8	(0.9)	2.2	(1.1)	2.2	(0.8)	85	13,4
7+7	Rec.	1	06:25	(1:49)	06:27	(1:59)	2.0	(1.2)	2.1	(1.3)	3.0	(1.0)	3.2	(1.0)	2.2	(1.1)	2.7	(1.3)	2.4	(1.1)	87	7,5
7+7	Rec.	2	06:46	(1:55)	07:03	(1:58)	1.7	(0.9)	1.9	(1.2)	2.8	(1.0)	3.0	(1.0)	2.1	(1.0)	2.6	(1.3)	2.1	(0.9)	89	6,6
7+7	Rec.	3	06:40	(1:41)	06:45	(1:47)	1.6	(1.0)	1.7	(1.0)	2.7	(1.0)	2.9	(0.9)	1.9	(0.9)	2.5	(1.1)	2.1	(0.9)	87	7,8
7+7	Rec.	4	06:14	(1:28)	06:20	(1:33)	1.6	(0.9)	1.6	(0.9)	2.7	(1.0)	3.0	(1.0)	1.9	(1.0)	2.3	(1.2)	2.2	(1.0)	87	8,0
7+7	Rec.	5	06:33	(1:31)	06:47	(1:44)	1.6	(0.8)	1.6	(0.8)	2.7	(0.9)	2.8	(0.9)	1.6	(0.7)	2.3	(1.1)	2.0	(0.8)	88	7,4
7+7	Rec.	6	06:37	(1:15)	06:48	(1:32)	1.6	(0.8)	1.7	(0.9)	2.3	(0.9)	2.7	(1.1)	1.7	(0.8)	2.4	(1.2)	2.0	(0.9)	87	8,1
7+7	Rec.	7	07:06	(1:41)	07:09	(1:57)	1.5	(0.7)	1.7	(0.9)	2.5	(0.9)	2.5	(0.9)	1.8	(1.0)	2.4	(1.2)	2.0	(0.9)	87	5,8

Average (avg) and standard deviation (SD) of 26 days in the three different work schedules (2+2, 4+4 and 7+7) of consecutive night shifts followed by consecutive recovery (rec.) days based on 73 police officers. Difficulty falling asleep, sleep quality, disturbed sleep, premature awakening number of awakenings, difficulty of awakening, non-refreshing sleep were all measured on a scale ranging from 1-5 with higher scores representing poorer sleep. Primary sleep duration (PSD), total sleep time (incl. naps.; TST) and sleep efficiency were assessed by actigraphy.
